# Influenza: Environmental Remodeling, Population Dynamics, and the Need to Understand Networks

**DOI:** 10.3389/fpubh.2014.00153

**Published:** 2014-09-29

**Authors:** María Paula Ortiz-Rodriguez, Luis Carlos Villamil-Jimenez

**Affiliations:** ^1^Epidemiology and Public Health Group, Universidad de La Salle, Bogotá, Colombia

**Keywords:** hot spots, influenza, networks, disease dynamics, animal reservoirs

## New Challenges for Public Health

Emergence of new pathogens has been the reality of the 21st century; the role of animal reservoirs and changes in human behaviors may be the main key factors for disease dynamics. Human population growth and territory expansion have lead to habitat sharing between human beings, domestic animals, wild animals, and their pathogens; bringing new opportunities for spill over. Risk assessment regarding the main factors associated with potential reassortment and transmission between species should get to a stage where the analysis of wildlife networks and interactions with domestic animals and human beings is well mapped. This last will allow an accurate prevention and control of hot spots of influenza transmission (1, 2).

The challenge for all public health professionals lies upon the integration of the analysis of environment, animal reservoirs, and human population as a whole and develop action plans accordingly to their interactions and not each of them separately (3–5).

New animal production systems and human population dynamics have lead to different sources of infection that are not well understood. Although surveillance systems have improved considerably, the need for better communications and relations between environmental, health, and agricultural sectors is essential for a precise prevention of disease appearance and dispersal. Interdisciplinary and Intersectorial help becomes imperative when assessing the risk factors; such as human interactions with animals, wildlife contact with animal production systems, and environmental remodeling that contribute to disease emergence (6). In addition, the assessment of hot spots of influenza transmission should be the tool to map animal habitats that are at most risk of encounters with domestic animals that might serve as a mixing vessel and as the source of infection for humans (7, 8).

The emergence of influenza viruses is just one example of many diseases that have social and environmental factors that enhance their appearance and dispersal. The new strains that have emerged have social and environmental issues in common, which contribute to the appearance of new viruses, or at least, to the spillover between species; and it is here where the efforts should focus (9, 10).

## Multicasuality, Networks, and Disease Emergence – Interdiciplinary Call

The close interaction between human beings and animals has determined many social behaviors, food availability, and diseases present nowadays (11, 12). When humans domesticated animals they started to be in close contact not only with the animals but also with the pathogens that they hosted. Some of these pathogens may have been of low pathogenicity in animals but after they acquired the ability to infect humans, they became pathogenic and even fatal for some hosts. This last is true only for some viruses that have the molecular characteristics that allow them to jump from one species to another, where genetic rearrangements and mutations result in new strains that infect more than one host from different species (13–15).

Spill over is the term used when a pathogen acquires the ability to jump from one species to another, allowing it to move to other habitats, and finally establish within a new niche (16). Is in these new niches where animal networks should be well assessed in order to prioritize the risks areas and animals involved in the dispersal and transmission of influenza virus for instance. As a consequence, when these interactions are understood the whole marketing systems and live bird markets’ (LBMs) chains can be mapped and controlled (17, 18).

Understanding of the complete commerce chain; starting with the poultry farm and ending in the LBMs have been addressed by Martin and his colleagues. This sort of studies will be enriched if the contacts between wild and domestic animals are mapped to pinpoint the hot spots of possible niches where reassortment of the virus or an outbreak might take place (17, 19).

As it has been mentioned before, environmental interactions between animals, pathogens, and human beings play a crucial role in disease dynamics and its emergence or re-emergence. However, it is not only the environmental surroundings that determine the contact of this last three: social interactions, economic activities, and food related preferences and trade, but also have an impact and should be well assessed when conducting control and surveillance actions (16, 20). For influenza viruses, there are two main facts that should be well addressed. In the first place, the role of animal reservoirs such as migratory birds and bats and, in the second, the role of poultry farms, live animal markets, and how the animals are sold, transported, and maintained in these (16, 21, 22).

Yet the above reasons give rise to new research and partnership opportunities that will need the participation of many disciplines. For instance, these new challenges will allow the accurate integration of the one health concept in the new approach to disease prevention.

## Hot Spots and Influenza Transmission – The Key for Prevention

Network dynamics both in human beings and animals will determine the new pathogens for human populations (23, 24). Since climate change, population growth, and expansion are phenomena that are the reality for this century, human and animal health professionals will need to work from a population based perspective, but this time assessing the environment in which the interactions take place. Tracking the possible strategic spots in which intervention measures can be conducted. Diseases have unique characteristics, and although they may be well understood nowadays the lesson arises when even in the 21st century, we encounter disease threats that have complex behaviors and that are caused by multicasuality (3, 25).

For instance, it will be of much use to have complete knowledge of the health status of wild animals that live close to animal production systems and/or human living areas (9, 26). Consequently active surveillance should be coupled with a better understating about animal behavior, distance traveled by the birds and/or bats, nesting, and resting sites. It will be of much use to establish if the birds are migratory or resident, and if migratory map their networks in both living sites. Lastly, not only endangered species should be treated carefully but also included in this type of studies; furthermore, sampling should be accompanied by mapping and census (27, 28).

We will be achieving the correct introduction of the one health concept into the production systems, economic chains, and disease dynamics if we sum up all the relations that intervene in the dynamic of the disease. Finally interdisciplinary work will allow for a better and broader analysis of all the risk factors that put the health status of a country at risk. Finally we will not be prepared to respond to a pandemic event until the understanding of the interactions and influence of these in disease dynamics are incorporated into the prevention measures.

## Conflict of Interest Statement

The authors declare that the research was conducted in the absence of any commercial or financial relationships that could be construed as a potential conflict of interest.
